# A CRISPR/Cas9 Generated Bovine CD46-knockout Cell Line—A Tool to Elucidate the Adaptability of Bovine Viral Diarrhea Viruses (BVDV)

**DOI:** 10.3390/v12080859

**Published:** 2020-08-06

**Authors:** Kevin P. Szillat, Susanne Koethe, Kerstin Wernike, Dirk Höper, Martin Beer

**Affiliations:** Institute of Diagnostic Virology, Friedrich-Loeffler-Institut, Federal Research Institute for Animal Health, 17493 Greifswald-Insel Riems, Germany; Kevin.Szillat@fli.de (K.P.S.); Susanne.Koethe@fli.de (S.K.); Kerstin.Wernike@fli.de (K.W.); Dirk.Hoeper@fli.de (D.H.)

**Keywords:** bovine viral diarrhea virus (BVDV), pestivirus, escape mutant, CD46, E^RNS^, adaptation, CRISPR, knockout, MDBK, cell entry

## Abstract

Bovine viral diarrhea virus (BVDV) entry into a host cell is mediated by the interaction of the viral glycoprotein E2 with the cellular transmembrane CD46 receptor. In this study, we generated a stable Madin–Darby Bovine Kidney (MDBK) CD46-knockout cell line to study the ability of different *pestivirus A* and *B* species (BVDV-1 and -2) to escape CD46-dependent cell entry. Four different BVDV-1/2 isolates showed a clearly reduced infection rate after inoculation of the knockout cells. However, after further passaging starting from the remaining virus foci on the knockout cell line, all tested virus isolates were able to escape CD46-dependency and grew despite the lack of the entry receptor. Whole-genome sequencing of the escape-isolates suggests that the genetic basis for the observed shift in infectivity is an amino acid substitution of an uncharged (glycine/asparagine) for a charged amino acid (arginine/lysine) at position 479 in the E^RNS^ in three of the four isolates tested. In the fourth isolate, the exchange of a cysteine at position 441 in the E^RNS^ resulted in a loss of E^RNS^ dimerization that is likely to influence viral cell-to-cell spread. In general, the CD46-knockout cell line is a useful tool to analyze the role of CD46 for pestivirus replication and the virus–receptor interaction.

## 1. Introduction

The genus *Pestivirus* belongs to the family *Flaviviridae* and contains several veterinary-relevant virus species with major animal welfare and economic importance like *pestivirus A* and *B* (bovine viral diarrhea virus types 1 and 2, BVDV-1 and -2), *pestivirus D* (border disease virus, BDV) and *pestivirus C* (classical swine fever virus, CSFV) [1,2,3,4]. The host range of the classical pestivirus species includes cloven-hoofed animals. However, in recent years, a continuously growing diversity of pestiviruses has been seen worldwide with atypical pestiviruses being isolated from host species like rat, bat and whale [5,6,7,8].

Pestiviruses are positive-sense, enveloped, single-stranded RNA viruses with a genome size of 12.3–13 kb [9]. The genome encodes eight nonstructural and four structural proteins in a single open reading frame (ORF) [10], of which the glycoproteins E^RNS^, E1 and E2 play an important role in the initiation of BVDV uptake by the host cell [11,12]. It has been shown that E^RNS^ interacts with the cell surface heparan sulfate and E1–E2 heterodimers bind the cellular receptor CD46 and mediate clathrin-dependent endocytosis [11,13,14]. CSFV E^RNS^ interacts with the heparan sulfate of porcine cells and both CD46 and heparan sulfate, are major factors for the attachment of CSFV in vitro [15,16]. Interestingly, a shift to a dominant role of the E^RNS^-mediated binding in CSFV was connected with a drastic in vivo attenuation [15]. Furthermore, dimerization of E^RNS^ is an important virulence factor and abrogation leads to attenuation [17]. E^RNS^ also plays a role in the control of the activation of beta interferon induced upon viral infection by inhibiting the double stranded RNA-induced response of cells [18]. Heparan sulfate has been further described to be important for cellular binding of different viruses, e.g., Schmallenberg virus, hepatitis E virus and rabies virus [19,20,21,22].

The binding partner of the pestivirus envelope protein E2, cellular CD46, is a type 1 transmembrane glycoprotein expressed on all nucleated cells that protects host cells from damage by the complement system by the inactivation of C3b and C4b complement products (reviewed in [23]). CD46 is known to serve as a binding partner for several human pathogens like certain adenoviruses, as well as for animal viruses like BVDV and CSFV [16,24]. In particular the two peptide domains E_66_QIV_69_ and G_82_QVLAL_87_ of the complement control protein module 1 (CCP1) are crucial for the binding of BVDV—preincubation of MDBK cells with an anti-CD46 serum leads to a strong reduction in infection efficiency [25]. In vitro studies have shown that BVDV spreads by direct cell-to-cell transmission from infected to uninfected cells, even in the presence of neutralizing antibodies against the virus and also if CD46 receptors are blocked by antibodies [26].

Interestingly, there is to our knowledge no bovine CD46-knockout cell line available, allowing e.g., the analysis of the interaction of bovine pestiviruses with receptor molecules. Due to rapid developments in the field of CRISPR/Cas9-mediated gene editing, genetically modified in vitro models like knockout cell lines can be generated in a straightforward manner in a relatively short amount of time [27]. By using ribonucleoprotein (RNP) complexes, target-specific guide RNAs (gRNAs) are complexed with the Cas9 protein and transfected into the target cell. Unlike delivery of mRNA or DNA, RNP-mediated editing does not depend on the host cell for the synthesis of Cas9 and gRNAs. Furthermore, the RNP-based approach reduces the risk of off-target effects and cell death due to the shortened half-life of the proteins inside the target cell [28,29]. We used the CRISPR/Cas9 RNP approach in this study to knockout the cellular receptor CD46 in the Madin–Darby Bovine Kidney (MDBK) cell line to establish a stable in vitro model. We used the generated cell line to passage different BVDV type 1 and 2 strains to study adaptation mechanisms of these viruses in a CD46-negative cellular environment.

## 2. Materials and Methods

### 2.1. Cells and Viruses

All cell lines used in this study were obtained from the Collection of Cell Lines in Veterinary Medicine (CCLV) at the Federal Research Institute for Animal Health, Insel Riems, Germany (FLI). Cells were cultured in minimal essential medium (MEM), supplemented with 10% fetal calf serum (FCS), at 37 °C and 5% CO_2_. A Madin–Darby Bovine Kidney cell line (MDBK, RIE0261) was used to generate the CD46-knockout cell line, further referred to as CD46-MDBK. Bovine esophagus cells (KOP-R, RIE0244) were used to propagate and titrate virus stocks of the BVDV-1 strains Paplitz (1b, cytopathic), NADL (1a), D02/11-2 (1d) and BVDV-2 strain CS8644 (2a). All pestiviruses were obtained from the German National Reference Laboratory for BVD/MD (FLI).

### 2.2. Generation of a CD46-knockout Cell Line

#### 2.2.1. Transfection of MDBK Cells and Clone Selection

The CRISPR/Cas9 RNP-mediated editing approach was used in this study to knockout the BVDV binding domains E_66_QIV_69_ and G_82_QVLAL_87_ of the cellular receptor CD46 in MDBK cells. CRISPR RNAs (crRNAs) were designed to introduce double-strand breaks (DBS) inside the CCP1 domain, spanning these binding domains (overview in Figure S1). Suitability of the crRNAs was confirmed by CRISPOR [30] and CHOPCHOP [31]. The crRNAs (crRNA-1: GGCTTCATAGAGACAAATCT and crRNA-3: TCATACACAATCTGCTCCCC) and all transfection reagents were purchased from Thermo Fisher Scientific (Waltham, MA, USA). Annealing of the crRNAs with the trans-activating crRNA (tracrRNA) was done following the manufacturer’s protocol. MDBK cells were seeded one day prior to transfection and subsequently transfected according to the manufacturer’s instructions, using Lipofectamine CRISPRMAX Cas9 transfection reagents (Thermo Fisher Scientific) and TrueCut Cas9 Protein v2 (Thermo Fisher Scientific). The nontarget control cell line (NTC) was generated under identical conditions, using nontarget control gRNAs (Thermo Fisher Scientific). Single cell dilution and subsequent polymerase chain reaction (PCR) of the monoclonal cell colonies were used to screen for a cell clone with the intended deletion.

#### 2.2.2. Isolation of DNA, PCR and Sequencing

DNA was isolated using the QIAamp DNA Mini Kit (Qiagen, Hilden, Germany) according to the manufacturer’s instructions. The PCR to confirm the knockout was performed using the QuantiTect Multiplex-PCR Kit (Qiagen) in combination with CD46-specific primers (forward primer CD46_CCP1_F: 5′-GAT GCT GTC TCT TCC ATT TAC T-3′; reverse primer CD46_CCP1_R: 5′-GCC TGA ATG CAT GGC TAT CT-3′) and the following conditions: 15 min, 95 °C; 45× (60 s, 95 °C; 30 s 58 °C and 30 s 72 °C); 5 min, 72 °C; 12 °C storage. The PCR amplicon of the wild-type MDBK cell line is 545 nucleotides long and covers the entire CCP1 domain.

PCR products were analyzed by gel electrophoresis and bands were extracted from the gel using the QIAquick Gel Extraction Kit (Qiagen) according to the manufacturer’s instructions. Single cell colonies with the intended knockout mutation display a truncated amplicon with a size of around 400 nucleotides. The PCR gel extract was further sequenced using the IonTorrent platform, as described later.

### 2.3. Immunofluorescence (IF) Staining, Plaque Assay and Fluorescence-Activated Cell Sorting (FACS)

Expression of the CD46 receptor by NTC and CD46^-^MDBK was visualized by IF staining, using antibodies BVD/CA 26/2/5 and BVD/CA 17/2/1(1:16 dilution in tris-buffered saline with tween (TBST)). The antibodies are directed against CD46 and were kindly provided by Prof. Becher, Institute of Virology, University of Veterinary Medicine, Hannover [32]. Antimouse Alexa Fluor™488 F(ab’)2 (1:1000 in TBST, Life Technologies, Carlsbad, CA, USA) was used as conjugate. FACS and IF was used to study the impact of the CD46-knockout on pestivirus entry into the host cell. In brief, CD46^-^MDBK and NTC cells were seeded one day prior to infection. On the day of infection, the cells were incubated with the different pestivirus isolates for 1 h at 37 °C and 5% CO_2_, multiplicity of infection (MOI) of 1. Confluent cells were used at this point to study the initial infectivity of the virus isolates and at later time points the cell-to-cell spread. Uninfected cells were used as a negative control and incubated with maintenance medium only (supplemented with penicillin and streptomycin). After the incubation period, all cells were washed and cultured for 24 h at 37 °C and 5% CO_2_ in maintenance medium.

For the IF-staining of pestiviruses, cell supernatant was discarded, and cells were fixed at 80 °C for 2 h. Subsequently, fixed cells were incubated with a 1:500 dilution of antibody WB103/105 (APHA Scientific, Addlestone, UK) in TBST for 1 h at room temperature (RT). Cells were washed thrice with TBST and incubated with a 1:1000 dilution of antimouse Alexa Fluor™488 F(ab’)2 (Life Technologies) in TBST for 1 h at RT. Cells were washed thrice and finally covered with 1,4-Diazabicyclo [2.2.2]octane (DABCO) fluorescence preservation buffer (Sigma-Aldrich, St. Louis, MO, USA) containing propidium iodide (Sigma-Aldrich).

For the IF plaque assay, CD46^-^MDBK cells were seeded one day prior to infection into a 24-well plate. Subsequently, cells were infected with the stock virus (passage 0) and passage 15 of virus isolate Paplitz (BVDV-1b) at MOI = 0.1 and incubated for 1 h at 37 °C, 5% CO_2_. Thereafter, the cells were washed once with maintenance medium and overlayed with a 1:1 mix of 4% agarose (Lonza, Basel, Switzerland) in aqua dest. and MEM (2× concentrated) supplemented with penicillin/streptomycin and gentamicin. The medium layer was removed after 48 h, cells were washed once with TBST and fixed with 4% paraformaldehyde (Sigma-Aldrich) for 20 min. Cells were washed once and permeabilized with 0.5% Triton X-100 in phosphate-buffered saline (PBS) for 5 min. Cells were washed again and treated as previously described for the IF staining of pestiviruses. The size of 50 virus plaques was measured manually by using the Nikon Eclipse Ti-U inverted microscope (Nikon GmbH, Düsseldorf, Germany) and NIS-Elements software (v. 4.50). The assay was performed in triplicate. The difference between the groups was statistically evaluated with a Mann-Whitney rank sum test as implemented in SigmaPlot (Version 11.0; Systat Software Inc., San Jose, CA, USA).

For FACS analysis, cell supernatant and trypsinized cells were collected in a FACS tube (Sarstedt, Nümbrecht, Germany) and centrifuged for 5 min at 800× *g*. Supernatant was discarded and cells fixed with 4% paraformaldehyde (Sigma-Aldrich) for 15 min at 4 °C. Cells were further permeabilized with 0.01% Digitonin (Sigma-Aldrich) for 10 min at 4 °C and washed with PBS. Cells were incubated with antibody WB103/105 (1:500 dilution in PBS) for 15 min at 4 °C. The cells were washed with PBS prior application of the anti-mouse Alexa Fluor™488 F(ab’)2 (1:1000 dilution in PBS) for 15 min at 4 °C. Cells were washed and resuspended in PBS. A total of 10,000 events were acquired from each sample, using the FACSCalibur (BD Biosciences, Franklin Lakes, NJ, USA). Uninfected cells were stained and used as a negative control. They were included in each run separately to allow subtraction of the background signal (<2.0%). Experiments were performed in three independent replicates.

### 2.4. Pestivirus Passaging

The different pestivirus isolates were passaged on CD46^-^MDBK and NTC cells to study the ability of the viruses to escape CD46-dependent cell entry and as a control, respectively. Cells were seeded one day prior to infection into a 24-well plate and infected with passage 0 (MOI = 1) for 1 h at 37 °C and 5% CO_2_. Passage 0 represents the virus stock grown from the initial virus isolates on KOP-R cells. Cells were washed once and incubated in 1 mL maintenance medium for 72 h before they were frozen at −20 °C. For the next virus passage, crude cell extract (noncleared mix of cells and supernatant) were thawed and 100 µL per well were used for infection of the next passage of CD46^-^MDBK and NTC cells. Virus titers of passage 15 were determined by endpoint titration on KOP-R cells and further tested in FACS and IF regarding their infectivity on CD46^-^MDBK.

### 2.5. Sequencing, Sequence Assembly and Sequence Comparison

Virus passages 0 and 15 of the virus isolates passaged on CD46^-^MDBK were processed using a modification of the protocol published by Wylezich and colleagues [33] and sequenced using the Ion Torrent S5XL platform. Briefly, RNA was extracted from the freeze-thawed crude cell extract using the RNAdvance kit (Beckman Coulter, Fullerton, CA, USA) following the manufacturer’s instructions and further concentrated using the Agencourt RNAClean XP magnetic beads (Beckman Coulter). Afterwards, cDNA was synthesized using a combination of the SuperScript™ IV First-Strand cDNA Synthesis System (Thermo Fisher Scientific) and the NEBNext^®^Ultra™ II Non-Directional RNA Second Strand Synthesis Module (New England Biolabs, Ipswich, MA, USA). The generated cDNA was fragmented to 500 bp using the Covaris M220 Focused-Ultrasonicator (Covaris, Brighton, UK) before Ion Torrent-compatible libraries were generated using the GeneRead L Core Kit (Qiagen) and IonXpress Barcode Adapter (Thermo Fisher Scientific). After size selection, library quality was checked using the Agilent 2100 Bioanalyzer system (Agilent Technologies, Santa Clara, CA, USA). The library concentration was measured with the KAPA Library Quantification kit (Roche, Mannheim, Germany). Using an Ion Torrent S5 XL, libraries were sequenced on an Ion 530 chip in 400-bp mode according to the manufacturer’s instructions (Thermo Fisher Scientific).

For sequence assembly, a random subset of 50,000 to 500,000 reads was assembled (Newbler v.3.0; 454/Roche). The complete data set was mapped against the assembled genome (Newbler v.3.0; 454/Roche) to confirm the sequence. Consensus sequences were annotated based on the NADL (BVDV-1a) reference strain (NC_001461) and amino acid (aa) sequences were annotated accordingly (E^RNS^: aa 271-497, E1: aa 498-692 and E2: aa 693-1066). Sequences were aligned using the MAFFT alignment of the bioinformatic software Geneious Prime (v.2020.1.2; Biomatters Ltd., Auckland, New Zealand). The variant analysis for the detection of viral quasispecies was performed in Geneious Prime (v.2020.1.2; Biomatters Ltd., Auckland, New Zealand), using the 454Contigs.bam from the mapping of passage 0 and passage 15 reads against the consensus sequence of passage 0 (Newbler v.3.0./Roche). Single-nucleotide-polymorphisms were detected with a minimum variant frequency of 0.1.

### 2.6. Immunoblotting

Cell lysates of passage 15 (on CD46^-^MDBK cells) and passage 0 (on NTC cells) were prepared 72 h after infection of the cells. For this purpose, cells were harvested and incubated for 30 min on ice in lysis buffer (Na_2_HPO_4_, 1% Triton), supplemented with protease inhibitor (Roche). Proteins were separated under nonreducing conditions on a 10% SDS-polyacrylamide gel for 75 min at 130 mA and subsequently blotted onto a GE Healthcare Amersham™ Protan™ nitrocellulose membrane (Thermo Fisher Scientific). The membrane was blocked with Roti^®^Block (Carl Roth, Karlsruhe, Germany) for 1 h. For the detection of pestivirus E^RNS^, the membrane was incubated with monoclonal antibodies HC/TC169/2/3 and BVD/C46/2/1 for 1 h (1:100 in Roti^®^Block, kindly provided by Prof. Becher, Institute of Virology, University of Veterinary Medicine Hannover). The POD-antimouse antibody (Dianova, Hamburg, Germany) was used as a secondary antibody, diluted 1:20,000 in phosphate buffered saline with tween (PBST). Proteins were detected by chemiluminescence, using SuperSignal™ West Pico PLUS Chemiluminescent Substrate (Thermo Fisher Scientific).

## 3. Results

### 3.1. Characterization of CD46^-^MDBK

**Figure 1:**
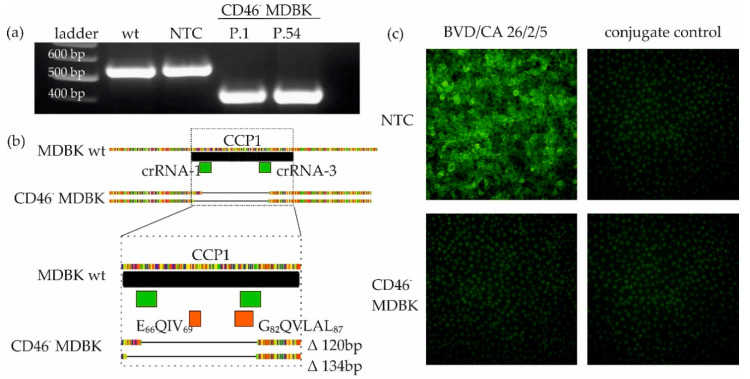
Characterization of CD46^-^MDBK cell line. (**a**) PCR amplification of knockout target region of MDBK wild-type (wt), nontarget control (NTC), CD46^-^MDBK passage 1 (P. 1) and passage 54 (P. 54) (**b**) Sequence comparison of wt and CD46^-^MDBK reveals a biallelic deletion of 120 and 134 nucleotides in the CCP1 region (black box), including the bovine viral diarrhea virus (BVDV) binding sides E_66_QIV_69_ and G_82_QVLAL_87_ (orange box). crRNAs are depicted in green (**c**) Staining of CD46 receptor using the anti-CD46 BVD/CA 26/2/5 antibody and conjugate only control on NTC (top) and CD46^-^MDBK (bottom). Magnification: 20×.

After single cell dilution of the transfected cells, colonies were screened via PCR and the knockout cell was identified to carry a homozygous deletion in the target region. Deep sequencing of the PCR product confirmed the successful biallelic knockout of 120 and 134 nucleotides in the target region, spanning the BVDV binding domains E_66_QIV_69_ and G_82_QVLAL_87_ (Figure 1b). CD46^-^MDBK were propagated and the deletion was confirmed by PCR up to the last passage used for passaging of the virus isolates (CD46^-^MDBK passage 54) (Figure 1a). IF staining with the anti-CD46 antibodies BVD/CA 26/2/5 and BVD/CA 17/2/1 (data not shown) showed no fluorescence signal on CD46^-^MDBK, compared to NTC cells (Figure 1c).

**Figure 2:**
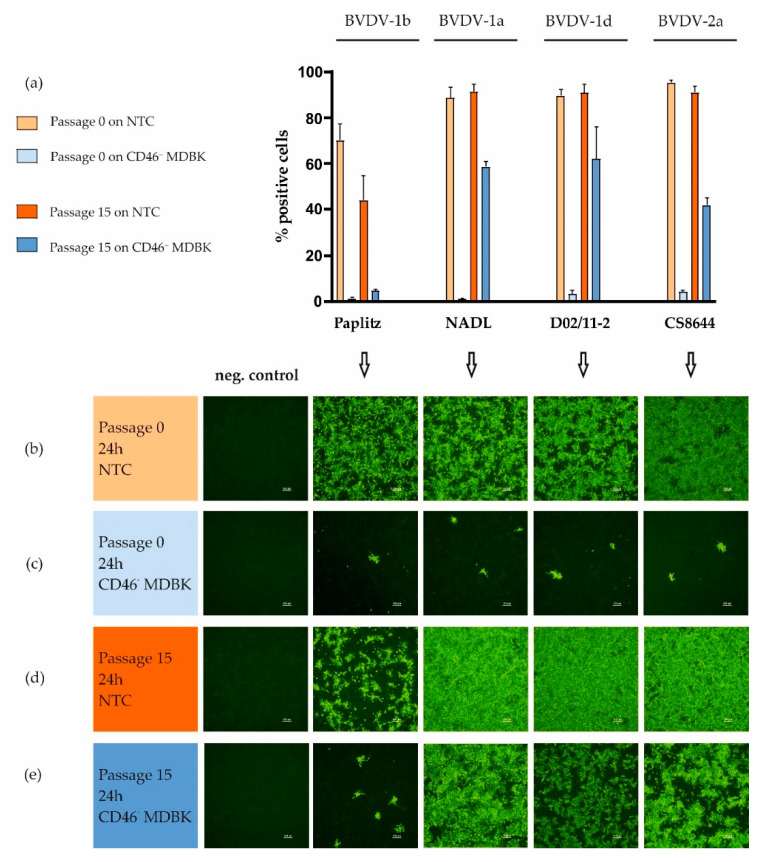
Detection of BVDV in the initial and the 15th passage on the indicated cell lines after incubation for 24 h. (**a**) Diagram depicting the percentages of positive cells in the fluorescence-activated cell sorting (FACS) analysis, conducted in triplicates. Immunofluorescence (IF) staining of (**b**) isolate passage 0 on non-target control (NTC), (**c**) passage 0 on CD46^-^MDBK (**d**) passage 15 on NTC and (**e**) passage 15 on CD46^-^MDBK. Scale bar 100 µm. Cells were incubated with the different pestivirus isolates (MOI = 1) for 1 h at 37 °C and subsequently washed and incubated for 24 h before analysis. Negative control: cells were incubated with maintenance medium only.

The functional knockout of the receptor was further confirmed via FACS and IF. The different BVDV isolates grew successfully on the NTC cells, whereas growth was strongly inhibited on the CD46^-^MDBK cells (Figure 2b,c). Based on the FACS data, the isolates used in this study (passage 0) infected 70–95% of the NTC cells, compared to less than 5% of the CD46^-^MDBK (Figure 2a).

### 3.2. Adaption of Pestivirus Isolates by Passaging on CD46^-^MDBK Cells

The generated CD46^-^MDBK cell line was subsequently used to adapt different BVDV isolates to a host cell lacking the CD46 receptor. After 15 passages on the knockout cell line, the isolates NADL, D02/11-2 (BVDV-1d) and CS8644 (BVDV-2a) showed a strong increase in infectivity, from less than 5% positive cells (passage 0) to 40–60% (passage 15) on CD46^-^MDBK as measured by FACS analysis (Figure 2a). For all viruses, infectivity analyzed by IF staining was in line with the FACS data (Figure 2).

**Figure 3:**
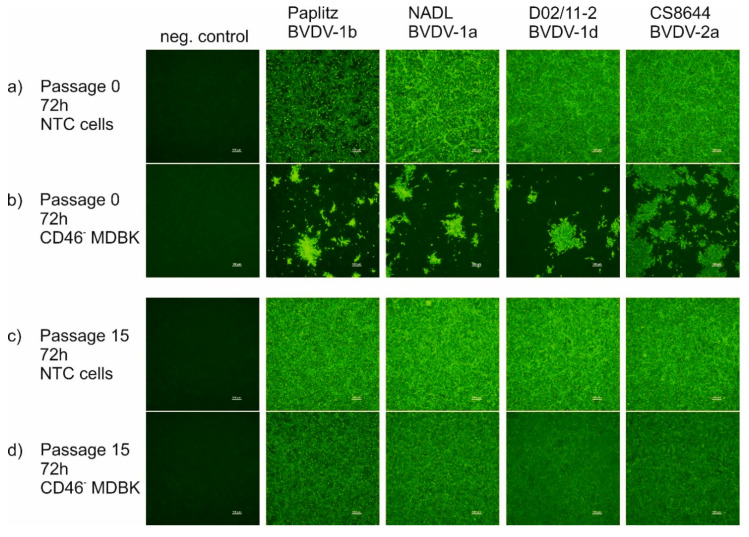
Comparison of the initial passage and passage 15 on non-target control (NTC) and CD46^-^MDBK after incubation for 72 h. Passage 0 on (**a**) NTC and (**b**) CD46^-^MDBK, passage 15 on (**c**) NTC and (**d**) CD46^-^MDBK. Cells were incubated with the designated pestivirus isolates (MOI = 1) for 1 h at 37 °C and subsequently washed and incubated for 72 h before analysis. Negative control: cells were incubated with maintenance medium only.

The cytopathic virus isolate Paplitz (cytopathic effect becomes apparent 3 to 5 days post infection) did not show a significant increase in CD46^-^MDBK cell infectivity after passaging based on the FACS results (from 1% positive cells with passage 0 virus to 4% with passage 15 virus, Figure 2a). Similarly, the IF staining did not show a strong increase in infectivity after incubation for 24 h on CD46^-^MDBK, when compared to the other isolates (Figure 2e). However, when the CD46^-^MDBK cells were incubated for 72 h, a complete infection of the cell layer was visible for all isolates (passage 15) (Figure 3d), that differed markedly from incubation for 72 h with the passage 0 virus (Figure 3b).

**Figure 4:**
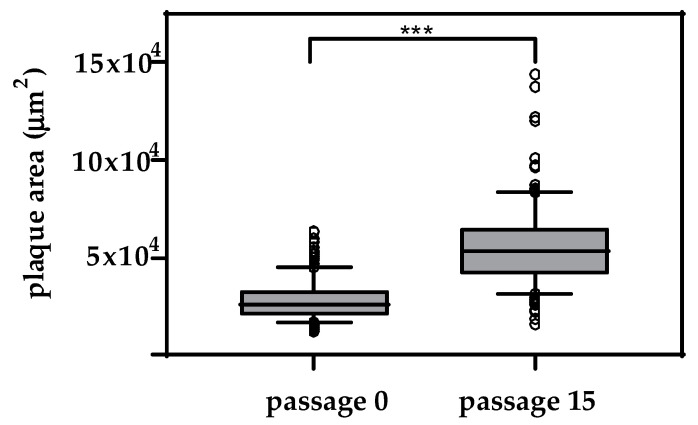
Comparison of plaque sizes of the initial passage and passage 15 of virus isolate Paplitz (BVDV-1b) on CD46^-^MDBK. Cells were incubated with a MOI = 0.1 for 1 h at 37 °C and subsequently washed and incubated for 48 h. The experiment was conducted in triplicate and 50 plaques were measured for each virus passage in each experiment (total *n* = 150 plaques/isolate). Whiskers indicate the 10% and 90% percentiles, respectively, and each outlier is depicted by a dot. The difference between the plaque sizes is statistically significant (*** *p* < 0.001).

Therefore, passage 0 and 15 of virus isolate Paplitz (BVDV-1b) were further tested in a plaque assay, to investigate the underlying mechanism that led to a densely infected cell layer after 72 h. Virus plaques of passage 0 had an average area of 28,462 µm^2^ (standard deviation (SD): 11,230 µm^2^, median: 25,489 µm^2^). Virus plaques of passage 15 were on average twice as big, with an average area of 55,985 µm^2^ (SD: 21,349 µm^2^, median: 53,725 µm^2^) (Figure 4).

The difference between the plaque sizes of both groups was statistically significant (*p* < 0.001). In comparison, the plaque size of the other passage 0 virus isolates measures for NADL (BVDV-1a): 25,243 µm^2^ (SD: 11,850 µm^2^, median: 23,132 µm^2^), D02/11-2 (BVDV-1d): 24,655 µm^2^ (SD: 9181 µm^2^, median: 24,011 µm^2^) and for CS8644 (BVDV-2a): 52,072 µm^2^ (SD: 21,839 µm^2^, median: 50,417 µm^2^). Based on these measurments, the Paplitz (BVDV-1b) passage 0 isolate is comparable to the plaque size of the other tested BVDV-1 strains.

### 3.3. Sequence Analysis after Virus Passaging on CD46^-^MDBK

In order to identify genetic adaptations that led to the observed shift in infectivity and virus-growth characteristics between the different virus passages, they were sequenced using next-generation sequencing. The genomic regions coding for the E^RNS^, E1 and E2 proteins of the initial virus passage were compared to the 15th passage, since these regions are known to be involved in pestivirus entry into the host cell [11,14]. The graphical overview of the aligned sequences is depicted in Figure S2. After 15 passages, all virus isolates gained at least one aa substitution in the E^RNS^-protein (Table 1).

Virus isolates NADL (BVDV-1a), D02/11-2 (BVDV-1d) and CS8644 (BVDV-2a) displayed an aa exchange at the same position, namely 479 in the E^RNS^. NADL (BVDV-1a) and CS8644 (BVDV-2a) exchanged the uncharged glycine with a charged arginine and D02/11-2 (BVDV-1d) exchanged the uncharged asparagine with a charged lysine. The cytopathic strain Paplitz (BVDV-1b) exchanged an uncharged cysteine for a charged arginine at position 441 in the E^RNS^ (Table 1). From the 15 mutations listed, two were already detected in the viral sequence reads from passage 0 as a minor population. Amino acid mutation in NADL (BVDV-1a) at position 677 and in D02/11-2 (BVDV-1d) at position 479 were identified with a sequence prevalence of 36% and 42%, respectively (Table 1). An important finding is that the minor population at aa position 479 of D02/11-2 (BVDV-1d) passage 0 is linked with another minor population in passage 0 that substitutes the original lysine at position 480 with an asparagine with a frequency of 39.9% (Table S1). During analysis of the actual reads of passage 0, it becomes apparent that reads are coding either for a lysine-asparagine or an asparagine-lysine (aa position 479 and 480) but not for a lysine-lysine or asparagine-asparagine (visualized in Figure S3).

### 3.4. E^RNS^ Dimerization of Virus Isolate Paplitz (BVDV-1b)

**Figure 5:**
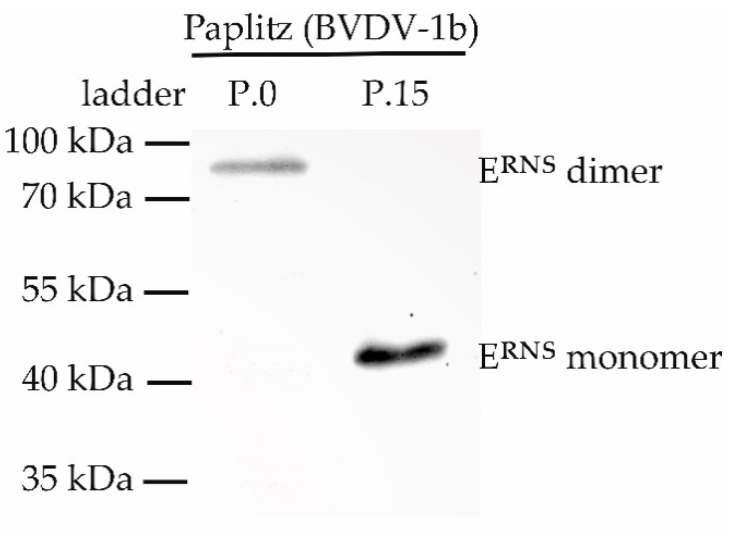
E^RNS^ formation of virus isolate Paplitz (BVDV-1b). Detection of E^RNS^ in the lysates of infected cells 72 h post infection using immunoblotting. Proteins were separated under nonreducing conditions. E^RNS^ of passage 0 (P.0) forms dimers (88–96 kDa), whereas passage 15 (P.15) forms monomers (44–48 kDa).

The formation of E^RNS^ dimers of virus isolate Paplitz (BVDV-1b) was analyzed, since the exchanged aa cysteine at position 441 was previously described to be important for dimerization of E^RNS^ [17]. Immunoblot analysis confirmed formation of E^RNS^ dimers (88-96 kDa) in virus passage 0, compared to monomer formation (48 kDa) of virus passage 15 (Figure 5).

## 4. Discussion

The importance of bovine CD46 for pestivirus entry into a host cell has been already described in detail in the past and the two peptide domains E_66_QIV_69_ and G_82_QVLAL_87_ have been identified to be crucial [25,34,35]. However, for further analysis, bovine CD46-knock-out cells were needed. We therefore successfully knocked out the aforementioned domains and showed that this leads to a significant decrease in infectivity of the tested BVDV-1 and -2 isolates.

The strong reduction in infectivity on CD46^-^MDBK is consistent with previous findings of Krey et al. [25], who showed reduced binding of pestivirus isolates in vitro by at least 70% (in 29 out of 30 isolates tested) after preincubation of MDBK cells with an anti-CD46 serum. Interestingly, we could show that the knockout of CD46 did not result in a complete loss of infectivity. A small percentage of less than 5% of the cells were still able to get infected by the tested BVDV isolates (passage 0). Therefore, a small fraction of the viruses most likely uses a CD46-independent route to enter the respective host cells.

After passaging the four different virus isolates 15 times on CD46^-^MDBK, infectivity of the virus isolates NADL (BVDV-1a), D02/11-2 (BVDV-1d) and CS8644 (BVDV-2a) increased significantly when compared to passage 0 after incubation for 24 h. Sequencing of these passages showed that the isolates share the same aa exchange at position 479 after passaging. This aa exchange is of importance, since the same position (aa 476 in *pestivirus C* (CSFV) correlates to aa 479 in *pestivirus A/B*) has been shown to be crucial for CSFV interaction with membrane-associated heparan sulfate [36]. The exchange of an uncharged aa (glycine) to a positively charged aa (arginine) at this position has been described to increase virus replication in vitro of CSFV variants carrying this mutation [15,36]. It has been also suggested that this aa substitution (position 476 in CSFV; position 479 in BVDV-1 and -2) increases the positive charge of the E^RNS^ region and that this particular aa is exposed to the surface and involved into direct binding to the negatively charged heparan sulfate [36]. Reimann and colleagues [37] also associated the increased virus infectivity in vitro with the same aa substitution of glycine to arginine at position 479 in CP7_E2alf, a chimeric pestivirus constructed from a BVDV-1 backbone (strain CP7) and E2 from CSFV (strain Alfort). Overall, these findings indicate very clearly that the loss of the host cells CD46 favours the introduction of a positively charged aa at position 479 of the E^RNS^ in the pestivirus isolates studied. Even though the aa position 479 is in the amphipathic C-terminal end of the E^RNS^ that is important for membrane binding, it is likely that this aa substitution has no or only minor effects on lipid binding. Research has shown that the amphipathic helix has a robust lipid affinity that cannot be disturbed by mutating single amino acids [38].

The aa substitution at position 479 occurred most likely de novo in virus isolate NADL (BVDV-1a) and CS8644 (BVDV-2a), however, very low frequencies might be missed due to the sequencing depths. In case of virus isolate D02/11-2 (BVDV-1d), the minor variant of the aa substitution (asparagine for lysine) at position 479 can be detected already in passage 0 with a proportion of 42%. Therefore, we would expect the phenotype of this passage 0 virus isolate to show a higher infectivity than actually detected, since we postulate that this mutation is important for the change to a phenotype with higher infectivity. However, when we looked into the other minor variants of passage 0 virus isolate D02/11-2 (BVDV-1d), we found that a lysine at aa position 480 is substituted with an asparagine with a similar proportion of 39.9%. Furthermore, when looking into the actual reads of passage 0, we could see that the reads are coding either for a lysine-asparagine (in consensus) or an asparagine-lysine (in minor variant) at position 479–480 but not for a lysine-lysine or an asparagine-asparagine. We therefore assume that in case of D02/11-2 (BVDV-1d), both lysine at position 479 and 480 are necessary to increase the infectivity of the virus. This has probably been selected for since neither the lysine-asparagine nor the asparagine-lysine variant could be detected in passage 15.

We suggest that the aa exchange at position 479 may allow the virus isolates NADL (BVDV-1a), D02/11-2 (BVDV-1d) and CS8644 (BVDV-2a) to compensate for the loss of the potential binding side CD46 by an increased binding of heparan sulfate. This finding provides therefore additional evidence for previous assumptions concerning the role of the heparan sulfate binding and CD46 independent entry of pestiviruses [25]. Furthermore, it would be interesting to passage these mutated virus isolates (passage 15) in vivo, to study if the mutation at position 479 is selected against in vivo like it was shown for CSFV [15]. We would speculate that this is the case, considering the similarities of BVDV and CSFV cell entry in vitro regarding their dependency on CD46 and heparan sulfate [11,16,34].

Nevertheless, the cytopathic strain Paplitz (BVDV-1b) is of special interest, since the virus shows an altered and unexpected growth behaviour after passaging. It seems that the selection pressure from CD46-MDBK cell passaging does not result in an improved initial cell entry of the virus but rather changed the growth behaviour of the virus towards a markedly improved cell-to-cell spread after initial cell entry. The lower infectivity of Paplitz (passage 15) compared to Paplitz (passage 0) on the NTC cells might be explained by the special way of adaption of this strain to the CD46 deficient environment and the subsequent loss of E^RNS^ dimers. It can be hypothesized that this trade-off favours cell spread in CD46-deficient cells for the cost of reduced binding to CD46 and growth in NTC cells. Interestingly, one mutation identified in the E^RNS^ of Paplitz (BVDV-1b) results in the exchange of a cysteine at position 441, which is important for the formation of a intermolecular disulphide bond in the E^RNS^ of CSFV [17]. Mutation of this cysteine, that occurred most likely de novo, leads to a loss of dimerization of E^RNS^ that results in case of CSFV in an attenuated virus that is still able to grow in cell culture [17] but also with larger plaque sizes due the reduced binding to heparan sulfate [39]. We could also demonstrate here the loss of E^RNS^ dimerization in the passage 15 variant. Paplitz (BVDV-1b) passages 0 and 15 are not able to enter the host cell faster after passaging, but once inside the cell, the virus grows differently than the initial passage 0 and shows an enhanced cell-to-cell spread. This change is likely influenced by substitution of the cystein at position 441, that leads to monomeric E^RNS^. Further research should study if the adaption described for virus isolate Paplitz (BVDV-1b) is typical for cytopathic pestiviruses and if the other mutations identified in E1 and E2 might also play a role. It could be hypothesized that dimerizaton of E^RNS^ could have a function concering cell-to-cell spread of pestiviruses like BVDV-1.

In summary, we describe here the succesful generation of a stable CD46-knockout cell line and we demonstrated that the infectivity of different BVDV isolates strongly depends on CD46. At the same time, we showed that a fraction of the virus particles is still able to enter the host cell even in the absence of the CD46 receptor. The study also shows that forced adaption of the pestiviruses studied leads to compensatory mutations in the E^RNS^, affecting the virus-host interplay. Forced adaption of virus isolates NADL (BVDV-1a), and CS8644 (BVDV-2a) and D02/11-2 (BVDV-1d) led to a mutation at aa position 479 probably increasing heparan sulfate binding. In contrast, isolate Paplitz (BVDV-1b) substituted a cystein at position 441 that led to a loss of E^RNS^-dimer formation, that is further suggested to increase cell-to-cell spread of the virus. The newly generated cell line is now available for future research to elucidate the role of the CD46 receptor in the context of the pestivirus biology and growth cycle.

## Figures and Tables

**Table 1:** Overview of the amino acid substitution in E^RNS^, E1 and E2 proteins based on the consensus sequences of the original BVDV isolates and their 15th passage on the CD46-deficient cell line. Amino acid positions with known effects are highlighted in bold.

Virus Strain	Amino Acid Position	Amino Acid Passage 0	Amino Acid Passage 15	Genome Region	Variant Frequency Passage 0	Variant Frequency Passage 15
Paplitz	**441**	**Cystein**	**Arginine**	**E^RNS^**	**-**	**98.7%**
(BVDV-1b)	673	Alanine	Valine	E1	-	54.3%
	764	Glycine	Arginine	E2	-	99.7%
NADL	450	Histidine	Leucine	E^RNS^	-	100%
(BVDV-1a)	**479**	**Glycine**	**Arginine**	**E^RNS^**	**-**	**100%**
	677	Isoleucine	Methionine	E1	36.0%	100%
D02/11-2	**479**	**Asparagine**	**Lysine**	**E^RNS^**	**42.1%**	**100%**
(BVDV-1d)	631	Alanine	Valine	E1	-	91.6%
CS8644	423	Glycine	Arginine	E^RNS^	-	75.3%
(BVDV-2a)	477	Glutamine	Lysine	E^RNS^	-	77.6%
	**479**	**Glycine**	**Arginine**	**E^RNS^**	**-**	**99.4%**
	480	Isoleucine	Threonine	E^RNS^	-	99.8%
	735	Histidine	Glutamine	E2	-	99.7%
	847	Isoleucine	Arginine	E2	-	99.4%
	880	Isoleucine	Valine	E2	-	95.8%

## Supplementary Materials

The following are available online at https://www.mdpi.com/1999-4915/12/8/859/s1. Supplementary Figure S1. Overview of the PCR amplicon, including the complement control protein 1 domain (CCP1, depicted in grey) with BVDV binding sides E_66_QIV_69_ and G_82_QVLA_87_ (both in orange), CRISPR RNAs (crRNA-1 and -3, depicted in yellow) and PCR primer (CD46_CCP1_F and _R, depicted in green). Supplementary Figure S2. Amino acid sequence alignment of passage 0 and 15 of the BVDV isolates. Differences between passages are depicted as a black gap in the alignment. The red box indicates the shared amino acid exchange at position 479 in virus passage 15 of NADL (BVDV-1a), D02/11-2 (BVDV-1b) and CS8644 (BVDV-2a). Supplementary Figure S3. Comparison of passage 0 and passage 15 sequence reads of D02/11-2 (BVDV-1b) at aa position 479 and 480 compared to consensus sequence passage 0. Two minor variants resulting in either a AAA-AAC or AAC-AAA codon (aa lysine-asparagine or asparagine-lysine). In comparison, passage 15 reads assemble only AAA-AAA (aa lysine-lysine) at position 479 and 480. The initial consensus sequence of passage 0 assembles AAC-AAA (aa lysine-asparagine). Supplementary Table S1. Overview of the variant analysis for passage 0 and 15 of virus isolate D02/11-2 (BVDV-1b). The variant of aa 479 and 480 is represented by nucleotide position 1437 and 1440 respectively and highlighted in bold.
