# Supplementary material for: A CRISPR/Cas9 Generated Bovine CD46-knockout Cell Line—A Tool to Elucidate the Adaptability of Bovine Viral Diarrhea Viruses (BVDV)
Source: Viruses. 2020 Aug 6;12(8):859. doi: 10.3390/v12080859 (PMC7472008; doi:10.3390/v12080859)
Supplement: Supplementary file 1 [file viruses-12-00859-s001.zip › Supplementary Table 1.docx]

| **Virus strain** | **Nucleotide position** | **Genome region** | **Nucleotide Change** | **Amino acid consensus** | **Amino acid variant** | **Frequency** |
| --- | --- | --- | --- | --- | --- | --- |
| Passage 0 | 867 | E^RNS^ | C → A | Alanine | Alanine | 40.4% |
| D02/11-2 | **1,437** | **E^RNS^** | **C → A** | **Asparagine** | **Lysine** | **42.1%** |
| (BVDV-1d) | **1,440** | **E^RNS^** | **A → C** | **Lysine** | **Asparagine** | **39.9%** |
|  | 1,802 | E1 | A → G | Glutamate | Glycine | 11.1% |
|  | 2,529 | E2 | T → A | Threonine | Threonine | 40.1% |
|  | 2,663 | E2 | G → A | Arginine | Glutamine | 38.6% |
|  | 2,754 | E2 | C → T | Cysteine | Cysteine | 13.8% |
|  | 2,830 | E2 | A → G | Threonine | Alanine | 13.5% |
|  | 2,837 | E2 | A → T | Tyrosine | Phenylalanine | 29.9% |
|  | 2,887 | E2 | G → A | Aspartate | Asparagine | 42.9% |
| Passage 15 | 943 | E^RNS^ | C → T | Leucine | Leucine | 89.2% |
| D02/11-2 | 1,224 | E^RNS^ | C → T | Cysteine | Cysteine | 92.4% |
| (BVDV-1d) | **1,437** | **E^RNS^** | **C → A** | **Asparagine** | **Lysine** | **100.0%** |
|  | 1,633 | E1 | C → T | Histidine | Tyrosine | 30.9% |
|  | 1,892 | E1 | C → T | Alanine | Valine | 91.6% |
|  | 1,962 | E1 | G → A | Threonine | Threonine | 13.3% |
|  | 2,508 | E2 | T → C | Asparagine | Asparagine | 90.1% |
|  | 2,529 | E2 | T → A | Threonine | Threonine | 10.1% |
|  | 2,704 | E2 | T → C | Tyrosine | Histidine | 34.8% |
|  | 2,940 | E2 | G → A | Glycine | Glycine | 91.1% |
|  | 3,073 | E2 | A → G | Threonine | Threonine | 14.2% |
|  | 3,082 | E2 | C → A | Histidine | Asparagine | 22.6% |
|  | 3,082 | E2 | C → T | Histidine | Tyrosine | 17.0% |
