# Supplementary figures and images for: A CRISPR/Cas9 Generated Bovine CD46-knockout Cell Line—A Tool to Elucidate the Adaptability of Bovine Viral Diarrhea Viruses (BVDV)
Source: Viruses. 2020 Aug 6;12(8):859. doi: 10.3390/v12080859 (PMC7472008; doi:10.3390/v12080859)

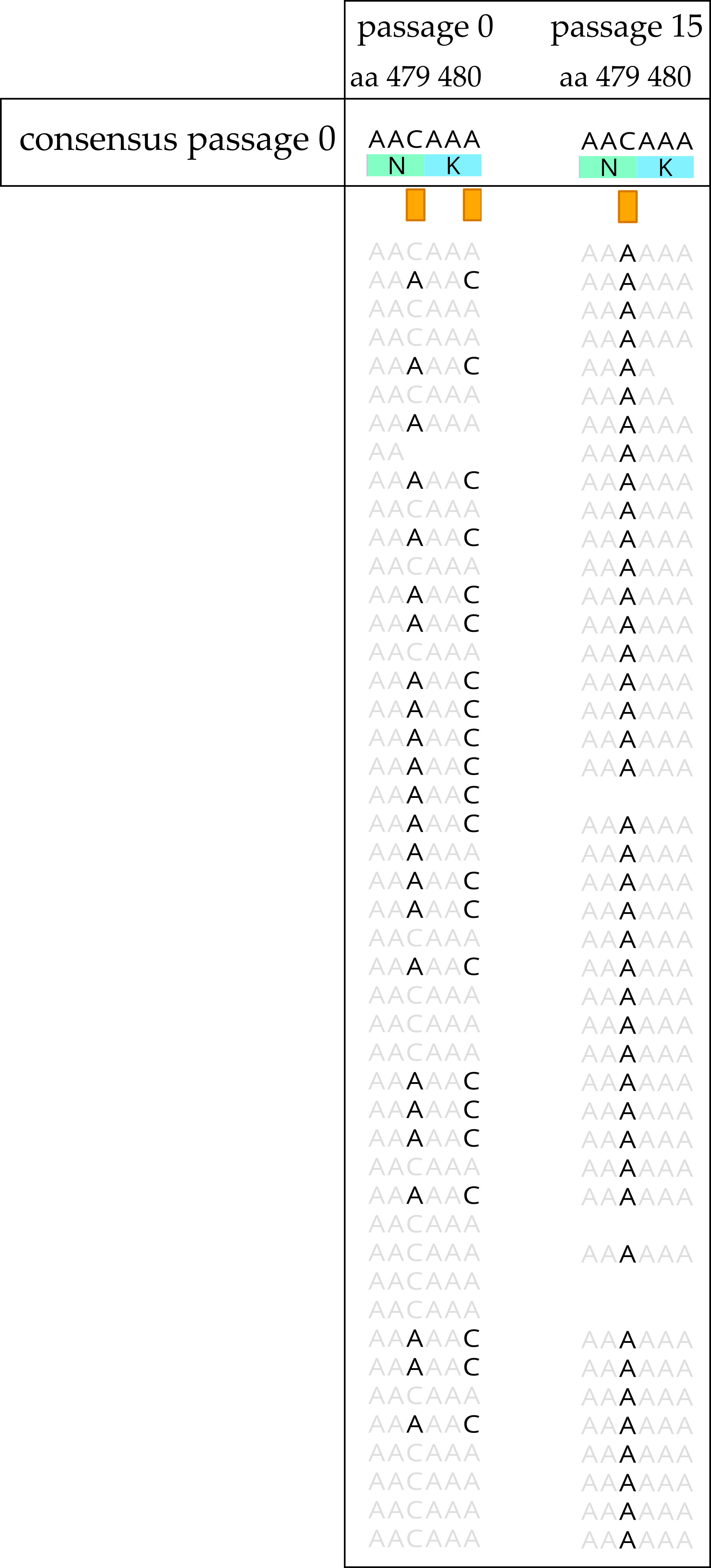

Supplement: Supplementary file 1 [file viruses-12-00859-s001.zip › Supplementary Figure 3.jpg]

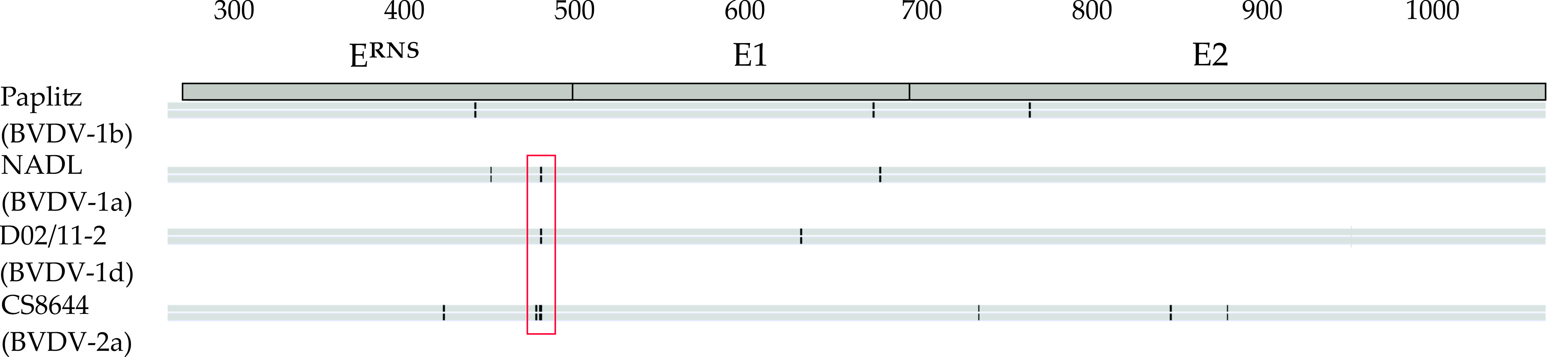

Supplement: Supplementary file 1 [file viruses-12-00859-s001.zip › Supplementary Figure 2.jpg]

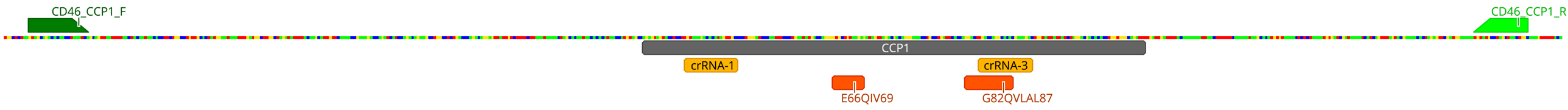

Supplement: Supplementary file 1 [file viruses-12-00859-s001.zip › Supplementary Figure 1.jpg]
